# Subcutaneus leiomyosarcoma of the neck: a case report

**DOI:** 10.1186/1757-1626-3-52

**Published:** 2010-02-03

**Authors:** Charalampos Skoulakis, Theognosia S Chimona, Paraskevi Tsirevelou, Chariton E Papadakis

**Affiliations:** 1 ENT Department, General Hospital of Volos, Greece; 2 ENT Department, General Hospital of Chania, Crete, Greece

## Abstract

**Introduction:**

Leiomyosarcomas are rare tumors. The most common site for head and neck leiomyosarcomas is the oral cavity, followed by sinonasal tract and skin. Subcutaneous leiomyosarcomas are thought to arise from small to medium-sized blood vessels in the subcutaneous tissue.

**Case Presentation:**

A 67-year-old female patient underwent excision of a slow growing neck mass of the left posterior neck triangle after a thorough clinical and laboratory examination. The lesion was located in the subcutis and fine needle aspiration biopsy revealed malignant features. Histology revealed subcutaneous leiomyosarcoma and the patient is free from local recurrence and distant metastases 3 years after wide excision of the lesion.

**Conclusions:**

The primary modality of therapy of subcutaneous leiomyosarcoma is surgery, adjuvant radiotherapy or chemotherapy may be used for control of local recurrence, in case of positive surgical margins, high-grade or large tumors.

## Introduction

Soft tissue sarcomas compromise approximately 0.7% of all malignant neoplasms, and leiomyosarcomas (LMSs) have been reported to account for 3-7% of soft tissue sarcomas [1]. LMSs are divided into those involving deep soft tissues sites as the retroperitoneum and those involving peripheral soft tissue sites. These peripheral soft tissue LMSs arising in either the dermis or the subcutis and together are referred to as superficial LMSs. Superficial LMSs arising in the dermis with or without extension into the subcutis are referred to as cutaneous LMSs while tumors arising in the subcutis are termed subcutaneous LMSs [2].

Subcutaneous LMS is thought to arise from small to medium-sized blood vessels in the subcutaneous tissue [3]. It is associated with higher rates of local recurrence, metastasis and death from disease, compared with lesions arising from cutaneous structures [3]. It usually presents as a painless or tender solitary subcutaneous nodule or group of nodules and has been reported to arise on the head and neck, back, thigh and beneath radiation dermatitis [4,5]. In this article, we report the case of a patient with a subcutaneous LMS located in the skin on the left side of the neck managed with wide surgical excision.

## Case report

**Figure 1:**
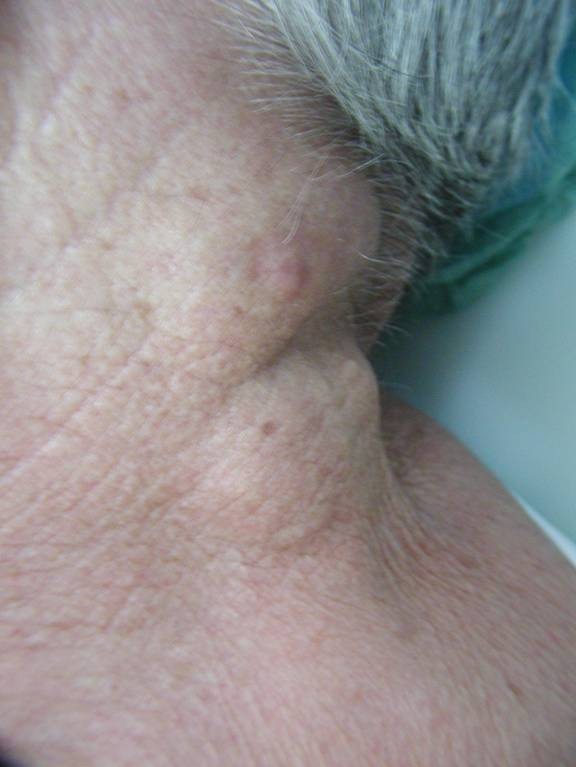
**The lesion on the left side of the patient's neck before excision**.

**Figure 2:**
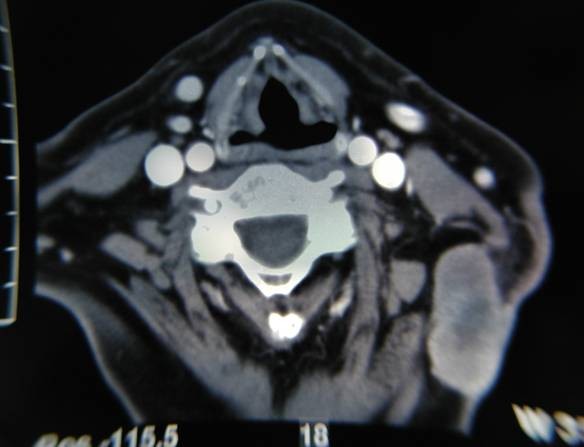
**Axial CT scan with contrast of the neck showing the lesion with peripheral enhancement and hypodense necrotic center in the subcutaneous tissue of the posterior neck triangle**.

A 67-year-old Greek woman, presented in the General Hospital of Volos with a painless neck mass on the left (Fig. 1). No previous operations were recorded in patient's medical history. She reported that the lesion appeared in the last year and had a slow growth. Fine needle aspiration biopsy (FNA) of the lesion revealed a small amount of inflammatory cells mainly lymphocytes and several neoplastic cells with malignant features advocating undifferentiating carcinoma. These cells were either scattered or aggregated with large, hyperdense nuclei, nuclear membrane grooves and several abnormal mitoses. Several neoplastic cells were spindle shaped with solid cytoplasm and multiple nuclei. Computed tomography with contrast of the neck region revealed a bi-lobe lesion of 3.2 × 2 × 4 cm dimensions with peripheral enhancement and hypodense necrotic center in the subcutaneous tissue of the posterior neck triangle with no involvement of the deeper muscle tissue (Fig. 2). After a thorough clinical examination, including endoscopy of the nasopharynx, larynx, and hypopharynx, and with no signs of metastatic disease, the lesion was excised under topical anesthesia.

**Figure 3:**
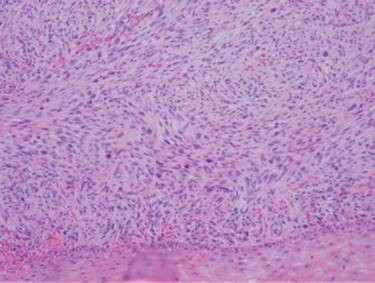
**Histology revealing perpendicularly arranged fascicles of spindle cells with eosinophilic fibrillary cytoplasm, scattered pleomorphic nuclei and irregular mitosis (Hematoxylin-Eosin ×200)**.

Histology showed a low grade nodular malignant mesenchymal neoplasm consisting of perpendicularly arranged fascicles of spindle cells with eosinophilic fibrillary cytoplasm, scattered pleomorphic nuclei and irregular mitosis with a rate of 6 to 8 per 10 high-power-fields (Fig. 3). The lesion was localized in the subcutaneous tissue, while medially it was in contact with striated muscle without infiltrating it. On immunohistochemical staining, the tumor expressed focal smooth muscle actin (α-SMA) and vimentin, while a small number of tumor cells were also weakly positive to HHF-35, desmin and S-100 stains. According to the histopathology and immunohistochemistry, the final diagnosis was subcutaneous LMS of the neck and the specimen's surgical margins were negative. Brain magnetic resonance as well as computed tomography of lungs and abdomen did not reveal any distant metastatic disease. Due to the local aggressive nature of the disease, the patient underwent a further wider local excision of the dermis and subcutaneous tissue of approximately 3 cm around the first excision and the defect was reconstructed with a regional rotational flap. No adjuvant therapy was recommended and three years postoperatively the patient is without signs of local recurrence or metastasis.

## Discussion

Soft tissue sarcomas are relatively rare neoplasms that may arise in any anatomic region. Occurrence in the head and neck accounts for less than 1% of all malignant tumors in this site [5]. Sarcomas of the head and neck most commonly present as painless submucosal or subcutaneous mass of uncertain duration. Superficial LMS is presented in middle age with a median age of diagnosis 45 to 50 years of age. A male-to-female predominance is reported of 2:1 to 3:1. Superficial leiomyosarcomas occur on the lower extremities (50-70%), the upper extremities (20-30%), the trunk (10-15%), and head and neck (1-5%) [3]. The most common site for head and neck LMSs is the oral cavity (22%), followed by sinonasal tract (19%) and skin (17%) [6]. Although subcutaneous LMS is thought to arise from small to medium-sized blood vessels in the subcutaneous tissue, it may originate from undifferentiated mesenchymal cells or it may be metastatic from other body regions, mainly as a late event associated with systemic metastasis and poor prognosis [6]. The low incidence of LMSs in the head and neck region is attributed to the scarcity of smooth muscle in this area which is limited to vessel walls, erector pili muscle of the hair follicles, esophagus and the posterior wall of the trachea [6,7]. Subcutaneous LMS may be covered with intact skin or may sometimes invade the overlying dermis. Management of these lesions should begin with a thorough clinical examination. Imaging studies, computed tomography (CT) and/or magnetic resonance imaging (MRI), usually follow the clinical evaluation and can be used individually or in combination. MRI has the advantage of delineating vascular involvement and is valuable for lesions located in the neck and parapharyngeal region. In case of a neck mass the appropriate work-up has to exclude the possibility of metastatic carcinoma or lymphoma. FNA in experienced hands can rule out metastatic squamous cell carcinoma, thyroid carcinoma or lymphoma. FNA results may also be suspected for soft tissue sarcoma, although it is difficult to diagnose a particular subtype of sarcoma. False negative results may be attributable to necrotic center of the tumor [5,7]. Subcutaneous LMS shows a higher primary growth rate than cutaneous LMS and a rate of local recurrence of 40 to 60%. Distant metastases affect commonly the lungs, bones, central nervous system and liver in 30 to 60% of patients [8]. Differential diagnosis includes myofibrosarcoma, fibrosarcoma, malignant nerve sheath tumor, malignant fibrous histiocytoma and rhabdomyosarcoma and can be made only after detailed histopathologic analysis [9].

Treatment of subcutaneous LMS is dictated by stage, location, size and patient age. The primary modality of therapy is surgery which must be designed to be curative. A wide excision with 3-5 cm lateral margins and a depth that includes subcutaneous tissue and fascia is recommended. Lymph node metastasis in head and neck sarcomas account for 10 to 15% of cases and neck dissection is not required for staging or treatment [5,6]. Radiotherapy has been used as an adjuvant therapy in order to minimize the incidence of local recurrence although sarcomas are considerable radio-resistant. Two indications for postoperative radiotherapy exist: 1) high-grade lesions and/or positive surgical margins, and 2) lesions larger than 5 cm and/or recurrence [10]. The outcome of chemotherapy in soft tissue sarcomas in head and neck is similar with that of the extremities, with adriamycin being the most important chemotherapeutic agent [7]. Although adjuvant therapy in LMSs is still controversial as no sufficient statistical evidence exists regarding their efficacy, chemotherapy combined with radiotherapy seems to improve local control of the disease especially when wide resection cannot be achieved [7,11]. A 42% recurrence rate for oral cavity and skin lesions has been reported [6]. Although tumor location and depth affect the recurrence rate and metastatic risk, the mitotic activity seems not to have a similar effect [11]. Head and neck sarcomas show worse survival outcomes compared with sarcomas of the extremities, with a 5-year survival rate between 49 and 55% [5]. Since lymph node metastasis is uncommon, the survival outcome depends mainly on local recurrence and distant metastasis control. Patients should be observed for a minimum of 5 years after surgery, as recurrence rates are variable, depending on the site of the tumor. Most of the patients present local recurrence within 2 years after initial management and those with recurrent disease are at risk for developing distant metastasis [8].

## Conclusion

Management of a neck mass may surprisingly reveal a rare lesion such as subcutaneous leiomyosarcoma. A thorough clinical and laboratory examination is essential in order to achieve fast diagnosis and curative excision of this aggressive tumor. The primary modality of therapy of subcutaneous LMS is surgery, which must be designed to be curative. Adjuvant radiotherapy or chemotherapy may be used for control of local recurrence in case of positive surgical margins, high-grade or large tumors.

## Abbreviations

LMS: leiomyosarcoma; FNA: fine needle aspiration; BMI: body mass index; α-SMA: smooth muscle actin; HHF-35: muscle-actin-specific monoclonal antibody; S-100: specific protein tumor-marker; CT: computed tomography; MRI: magnetic resonance imaging; HPF: high-power fields.

## Consent

Written informed consent was obtained from the patient for publication of this case report and accompanying images. A copy of the written consent is available for review by the Editor-in-Chief of this journal.

## Competing interests

The authors declare that they have no competing interests.

## Authors' contributions

CS and PT were the doctors that the patient presented to, performed the FNA as well as the surgical excision, TC and CP followed the patient in the postoperative period. All authors have discussed and make discissions for the treatment procedure followed. All authors have contributed in the manuscript writing. All authors read and approved the final manuscript.

## References

[B1] Gustafson P Willén H Baldetorp B Fernö M Åkerman M Rynholm A Soft tissue leiomyosarcoma. A population-based epidemiologic and prognostic study of 48 patients, including cellular DNA content Cancer 1992 70 114 119 10.1002/1097-0142(19920701)70:1<114::AID-CNCR2820700119>3.0.CO;2-U 1606532

[B2] Guillen D Cockerell C Cutaneous and subcutaneous sarcomas Clin Dermatol 2001 19 262 268 10.1016/S0738-081X(01)00177-8 11479038

[B3] Wascher R Lee M Recurrent cutaneous leiomyosarcoma Cancer 1992 70 490 492 10.1002/1097-0142(19920715)70:2<490::AID-CNCR2820700218>3.0.CO;2-F 1617597

[B4] Lin J Tsai R Subcutaneous leiomyosarcoma on the face Dermatol Surg 1999 25 489 491 10.1046/j.1524-4725.1999.08290.x 10469099

[B5] Pellitteri P Ferlito A Bradley P Shada A Rinaldo A Management of sarcomas of the head and neck in adults Oral Oncology 2003 39 2 12 10.1016/S1368-8375(02)00032-5 12457715

[B6] Suen J Vural E Waner M Myers E, Suen J, Myers J, Hanna E Unusual tumors Cancer of the head and neck 2003 Philadelphia: Saunders 611 629

[B7] Patel S Shaha A Shah J Soft tissue sarcomas of the head and neck: an update Am J Otolaryngol 2001 22 2 18 10.1053/ajot.2001.20699 11172210

[B8] Tran L Mark R Meier R Calcaterra T Parker R Sarcomas of the head and neck. Prognostic factors and treatment strategies Cancer 1992 70 169 177 10.1002/1097-0142(19920701)70:1<169::AID-CNCR2820700127>3.0.CO;2-F 1606539

[B9] Montgomery E Goldblum J Fisher C Leiomyosarcoma of the head and neck: a clinicopathological study Histopathology 2002 40 518 525 10.1046/j.1365-2559.2002.01412.x 12047762

[B10] Willers H Hug E Spiro I Efird J Rosenberg A Wang C Adult soft tissue sarcomas of the head and neck treated by radiation and surgery or radiation alone: patterns of failure and prognostic factors Int J Radiat Oncol Biol Phys 1995 33 585 593 7558947 10.1016/0360-3016(95)00256-X

[B11] Angeloni M Muratori F Marageli N Chalidis B Ricci R Rossi B Maccauro G Exophytic growth of neglected giant subcutaneous leiomyosarcoma of the lower extremity. A case report Int Semin Surg Oncol 2008 5 11 15 10.1186/1477-7800-5-11 18495007 PMC2409359

